# Conflict and cooperation in paranoia: a large-scale behavioural experiment

**DOI:** 10.1017/S0033291717003075

**Published:** 2017-10-17

**Authors:** N. J. Raihani, V. Bell

**Affiliations:** 1 Department of Experimental Psychology, University College London, London, UK; 2 Division of Psychiatry, University College London, London, UK; 3 South London and Maudsley NHS Foundation Trust, London, UK

**Keywords:** Delusion, game theory, psychosis, schizophrenia, social cognition

## Abstract

**Background:**

Paranoia involves thoughts and beliefs about the harmful intent of others but the social consequences have been much less studied. We investigated whether paranoia predicts maladaptive social behaviour in terms of cooperative and punitive behaviour using experimental game theory paradigms, and examined whether reduced cooperation is best explained in terms of distrust as previous studies have claimed.

**Methods:**

We recruited a large population sample (*N* = 2132) online. All participants completed the Green *et al.* Paranoid Thoughts Scale and (i) a Dictator Game and (ii) an Ultimatum Game, the former with an option for costly punishment. Following distrust-based accounts, we predicted highly paranoid people would make higher offers when the outcome depended on receiving a positive response from their partner (Ultimatum Game) but no difference when the partner's response was irrelevant (Dictator Game). We also predicted paranoia would increase punitive responses. Predictions were pre-registered in advance of data collection. Data and materials are open access.

**Results:**

Highly paranoid participants actually made lower offers than non-paranoid participants both in the Dictator Game and in the Ultimatum Game. Paranoia positively predicted punitive responses.

**Conclusions:**

These findings suggest that distrust is not the best explanation for reduced cooperation in paranoia and alternative explanations, such as increased self-interest, may apply. However, the tendency to attribute harmful intent to partners was important in motivating punitive responses. These results highlight differing motivations underlying adverse social behaviour in paranoia and suggest that accounts based solely on the presenting features of paranoia may need to be rethought.

## Introduction

Paranoia lies on a continuum, ranging from low-level paranoid thoughts to frank paranoid delusions that centre on concerns about others’ harmful intent (Freeman & Garety, 2000). Paranoia has typically been investigated using psychometric measures or tests that measure cognitive biases, reflecting the common focus on individualistic processes involved in evaluating personal threat (Freeman & Garety, 2014). However, relatively little research has explored social interaction despite adverse social behaviour being a common characteristic of paranoia (Freeman, 2016).

Paranoia strongly relates to processes involved in social decision-making and cooperation (Boyer *et al.* 2015; Patrzyk & Takáč, 2017) and there is now a nascent literature investigating paranoia using game theoretic paradigms. These paradigms involve social interactions where cooperative or non-cooperative decisions determine each participant's economic payoff (Camerer, 2003). These tasks are particularly relevant to paranoid concerns because they allow for cooperation and mutual benefit but also allow for harm, in the form of economic costs, and cheating, by gaining resources at the expense of the partner. These approaches therefore have the potential to reveal how paranoia affects social behaviour, and – because they are designed to selectively isolate specific social motivations – can help test and develop psychological theories.

### Paranoia and cooperative behaviour

Previous game theory studies report that paranoia predicts reduced cooperation in the Prisoner's Dilemma Game (Ellett *et al.* 2013) and reduced investments in the Trust Game (Fett *et al.* 2012, 2016; Gromann *et al.* 2013). These findings were interpreted as due to increased levels of ‘distrust’ in paranoia. Nevertheless, alternative explanations for reduced cooperation are possible. Reduced cooperation could reflect doubts about the partner's competence rather than fears about their intentions. Alternatively, it might stem from players’ increased desire to maximise their own payoffs (hereafter ‘self-interest’, used in the non-pejorative economic sense) since, in both the one shot Prisoner's Dilemma and Trust Games the individually payoff-maximising option is to defect (choose the non-cooperative option), regardless of how the partner behaves. To disentangle these motives, a comparison with a situation where the personal incentive to defect is still the same but where the partner's response is irrelevant in deciding payoffs is needed. In these so-called ‘non-strategic’ scenarios, where the partner's response is not a concern, self-interest rather than distrust is a more parsimonious explanation for reduced cooperation.

To test whether paranoia predicts reduced cooperative behaviour in the presence and absence of strategic concerns, we recruited participants to play the Dictator Game (Kahneman *et al.* 1986) and the Ultimatum Game (Güth *et al.* 1982). The Dictator Game is a non-strategic game where the ‘dictator’ decides how to split a pot of money with their partner (the ‘receiver’) who must accept any donation. Dictator donations therefore represent a relatively unbiased estimate of cooperative tendency in the absence of strategic concerns. In contrast, the Ultimatum Game is a strategic scenario where the responder decides whether to accept the proposer's offer – in which case both players are paid according to the proposed split, or can reject the offer – meaning neither player receives any money. To ensure a payoff, the proposer needs to judge what is likely to be an acceptable offer to the responder. These games provide a helpful contrast, allowing a test of how social decision-making differs in paranoia between two similar scenarios that vary only in whether one player needs to take into account the other's intentions when making a decision.

The Ultimatum Game also offers another method to disambiguate whether reduced cooperation stems from self-interest or distrust in a manner that is not possible in the Prisoner's Dilemma Game (where defecting could reflect either motive). Ultimatum Game rejections to unfair offers impose greater costs on the proposer than the responder, and leave open the possibility that the responder could be motivated by intent to harm the proposer. Indeed, previous research has shown that a perception that the responder will reject offers leads to more generous proposals (Slembeck, 1999). If reduced cooperation in paranoia stems from over-attributing harmful intentions (thereby reducing trust), we would expect paranoid participants to make more generous offers as proposers in the Ultimatum Game in order to counter what they perceive as higher levels of harmful intent in the responders. By contrast, if Ultimatum Game offers only reflect self-interest (i.e. where participants offer the lowest amount they expect the partner to accept), then we would not expect Ultimatum Game offers to vary with paranoia.

### Paranoia and punitive behaviour

Paranoid attributions of harmful intent should not only manifest in reduced cooperation but also increased punitive behaviour. Punishment occurs when one individual pays a cost to impose a reciprocal cost on a cheating partner (Raihani *et al.* 2012). Previous research has established that decisions to take punitive action involve a judgement of the severity of harm as well as a judgment of whether the harm was intended (Carlsmith *et al.* 2002; Cushman *et al.* 2009). A recent study confirmed that paranoia leads to increased levels of harmful intent attribution to partners in the Dictator Game where the actual motivation underlying dictator decisions is ambiguous (Raihani & Bell, 2017*a*). Therefore in this study, we expected these paranoid attributions to manifest behaviourally – by predicting an increased willingness to punish. We tested this prediction using two behavioural measures. In the Dictator Game, we introduced an unannounced opportunity for receivers to pay a small amount to impose a larger fine on the dictator after the dictator's decision. In the Ultimatum Game, rejecting offers can be interpreted as costly punishment since it involves incurring a cost to impose a larger cost on an unfair partner (Slembeck, 1999; but see Yamagishi *et al.* 2009). As such, we predicted paranoia would also predict increased rejection of Ultimatum Game offers.

### Study aims summary

We predicted highly paranoid people would make higher offers when the final outcome depended on a positive response from their partner (in the Ultimatum Game) but no difference when they believed that the partner's response was irrelevant (in the Dictator Game). We also predicted paranoia would increase punitive responses in the Dictator Game and rejections in the Ultimatum Game.

In contrast to studies which have recruited clinical participants with psychiatric diagnoses but have relatively low statistical power, we recruited a large sample that naturally includes people in the clinical range (Shapiro *et al.* 2013) and tested these predictions using analyses, which we pre-registered prior to collecting data.

## Methods

### Participants

This project was approved by the UCL Ethics Board (project 3720/001). Participation was voluntary and informed consent was obtained from all participants prior to taking part.

We recruited 3217 US-based participants to complete the Green *et al.* Paranoid Thoughts Scale (GPTS; Green *et al.* 2008) via the online crowdsourcing platform Amazon Mechanical Turk (MTurk, http://www.mturk.com). GPTS responses were collected in November–December 2016. Participants played two Dictator Games without punishment (Raihani & Bell, 2017*a*) in December 2016. These data were published in Raihani & Bell (2017*a*) and are not presented here. We successfully recalled 2132 (979 males; 1153 females) of the original 3217 participants in January–February 2017 to take part in the present study. Participants ranged in age from 18 to 80 years old (mean: 38.0 ± 0.26). Paranoia scores were unavailable for 11 participants, because the worker ID entered did not match any of the worker IDs in our existing database. Of the 2120 participants for whom we had paranoia scores, the mean score was 50.7 ± 0.49 (range: 32–160), compared with a mean of 48.8 ± 1.00 from the non-clinical sample in the original Green *et al.* (2008) study. The mean GPTS score for clinical patients with paranoid delusions in original study was 101.9; in our sample 108 participants (5%) scored above the Green *et al.* clinical mean (compared with 3% of non-clinical participants in the original Green *et al.* study).

Data were collected in two waves with a 15-day interval to reduce interference between tasks. In each wave, participants played both the Dictator Game and the Ultimatum Game (game order presentation was counter-balanced via random assignment across participants). In one wave, participants played as both the Dictator Game dictator and the Ultimatum Game proposer, while in the other wave they played as the Dictator Game receiver and the Ultimatum Game responder (see description below). The order in which participants were recruited to waves of the study was also counter-balanced. In each wave, participants received a payment of $0.20 for taking part, plus up to $1.10 depending on the outcome of the games. In total 1187 of the recalled participants took part in just one wave of the study, while 945 were successfully recruited to both waves.

### The experiment

In each wave, participants were truthfully informed that they would be assigned two different partners for each task, to reduce the potential for reciprocity or retaliation across the tasks. Most participants reported being confident or highly confident that they were interacting with a real partner (see online Supplementary Information). Participants made their decisions in isolation and partners were subsequently assigned via ex-post matching (c.f. Raihani *et al.* 2013). Using this method, decisions are stored and retrieved when the matched partner participates, and so although they involve genuine interaction, they do not occur in real time. Participants were required to correctly answer multiple-choice comprehension questions to ensure that they understood the contingencies of the different tasks (see online Supplementary Information for game instructions). Participants that failed a multiple-choice comprehension question were given a second attempt to answer correctly (using a free-form answer so that they could not select the correct option via trial and error). Participants who still answered incorrectly (46/1522, 3.0%, in the Dictator Game and 88/1544, 5.7%, in the Ultimatum Game answered at least one question incorrectly) could participate but we include incomprehension as a variable in analyses.

In the Dictator Game, participants were cast as either the dictator (in one wave) or the receiver (in the other wave). Note that loaded terms such as dictator and receiver were not used in the instructions seen by participants. Dictators were given $0.55 and informed that the receiver had been given $0.05. Dictators could then choose to send any division (from $0.00 to $0.55, in $0.05 increments) to the receiver. Importantly, dictators were not aware of the possibility that they could be punished until after they had made their donation. This was essential because we wished to collect a measure of generosity that was unbiased by strategic considerations. Receivers were informed of the starting bonuses of both players and the fact that the dictator could choose how much of a $0.55 starting bonus to send to them. Receivers could sacrifice $0.05 of their bonus to punish the dictator, by subtracting $0.15 from the dictator's bonus. Using the strategy method, receivers stated what donations from the dictator (from $0.00 to $0.55) they would punish – and were told that their decision would be executed based on the actual donation decision of the dictator. Previous studies have shown that this method is a reliable technique for eliciting behavioural responses in similar tasks (Brandts & Charness, 2011; Fischbacher *et al.* 2012), although it might yield conservative estimates of punitive tendency (Brandts & Charness, 2011).

In the Ultimatum Game, participants played as proposers in one wave and in responders in the other wave. Proposers were given $0.50 and instructed to offer any share of this endowment (from $0.00 to $0.50, in $0.05 increments) to the responder. Proposers were also informed that the responder had veto power over the division, such that if the responder rejected the offer then both players would get nothing. Responders were instructed to indicate which offers they would accept and which they would reject, using the strategy method as above. In doing so, we determined the responder's minimum acceptable offer (MAO). Responders were informed that their decision to accept or reject the proposer's offer would be implemented based on their MAO.

### Predictions

We specified eight *a priori* predictions, which were pre-registered before data collection on AsPredicted.org – https://aspredicted.org/mc5vz.pdf – and are included in the online Supplementary Information. We report one deviation for all analyses from the pre-registered analysis and two deviations related to data coding. The overall deviation was to include ‘failed comprehension’ as an explanatory variable in all models. Originally, we intended to exclude participants who failed comprehension checks but, to reduce the possibility of systematic bias from excluding participants, we used responses from all participants and controlled for the effect of failing at least one comprehension question in the analyses. All models were checked for robustness when failed comprehenders were excluded and discrepancies between the two approaches are reported.

Data coding deviations relate to the analyses for predictions 6 and 7 below and were due to non-monotonic strategies in the reported MAOs and punishment thresholds for some participants (*N* = 72/1551 in Ultimatum Game and 104/1551 in Dictator Game). Participants could display a non-monotonic strategy by, for example, stating that they would accept a proposer's offer of $0.20 but reject an offer of $0.50. Similarly, a non-monotonic punishment strategy would be implied if participants said they would punish a dictator donation of $0.20 but not $0.15. Non-monotonic MAOs in the Ultimatum Game can reflect advantageous inequity aversion (Brosnan & de Waal, 2014), though other explanations are also possible (e.g. Hennig-Schmidt *et al.* 2008). Since we did not anticipate non-monotonic strategies, we decided that a more conservative approach for P6 and P7 would be to assess whether participants rejected any non-zero proposer offer or punished any dictator donation (rather than analysing responses to all offers as originally envisaged).

### P1: Dictator Game donations will be lower than Ultimatum Game offers

To check the pattern of results in the whole sample matched established findings (Camerer, 2003), we ran a paired, two-tailed Wilcoxon signed rank test comparing Dictator Game donations with Ultimatum Game offers. *N* = 1529 participants.

### P2 & P3: Dictator donations will not vary with paranoia but Ultimatum Game offers will increase with paranoia

We ran two models, one with Dictator Game donation as the response term and one with Ultimatum Game offer as the response term. These response terms were each parameterised as a 7-level ordered categorical response term in two cumulative link models (CLM, package: ordinal; Christensen, 2015), with the terms ‘Age’, ‘Paranoia’, ‘Order’ (0 = played as DG dictator first; 1 = played as UG proposer first), ‘Failed Comprehension’ (0 = no comprehension questions wrong; 1 = at least 1 comprehension question wrong), ‘Gender’ (0 = female; 1 = male) and ‘Wave’ (1/2) set as potential explanatory terms. These response terms were included in all statistical models. Data were missing for seven participants, so both models are based on *N* = 1522.

### P4: Paranoia will be inversely associated with Dictator Game punishment threshold

Punishment threshold was defined as the highest dictator donation that the participant said they would punish. We had responses from 1544 participants. Of these, 859 participants who did not punish any donation were excluded, leaving 685 responses for analysis. As above, punishment threshold was a 7-level ordered categorical variable, with higher values indicating increased punitive tendency (i.e. a lower punishment threshold). We used a CLM to investigate the factors explaining the punishment threshold.

### P5: Paranoia will be associated with higher MAO in the Ultimatum Game

The MAO was specified as the minimum offer that a responder said they would accept in the Ultimatum Game. MAO was a 7-level ordered categorical variable and was set as the response term in a CLM. *N* = 1544 responses.

### P6: Paranoia will be positively associated with willingness to punish at least one dictator donation

Willingness to punish was a dummy variable where 0 = would not punish any dictator donation and 1 = would punish at least one dictator donation. This dummy variable was set as the response term in a Generalised Linear Model (GLM) with binomial error structure. *N* = 1544 responses.

### P7: Paranoia will be positively associated with tendency to reject at least one Ultimatum Game offer

We specified whether participants accepted all offers (=0) or rejected at least one offer in the Ultimatum Game (=1) and set this dummy variable as the response term in a GLM with binomial error structure. *N* = 1544 responses.

### P8: We do not know whether participants will be more willing to reject Ultimatum Game offers than they are to punish Dictator Game donations

To test whether the Ultimatum Game rejection threshold (defined as MAO – $0.05) was significantly different to the Dictator Game punishment threshold, we first converted these threshold amounts into proportions of the total stake, to account for the different stake sizes across the tasks ($0.55 and $0.50 in the Dictator and Ultimatum Game, respectively). We then ran a paired Wilcoxon signed rank test to see whether participants showed different thresholds for rejecting (Ultimatum Game) or punishing (Dictator Game) offers, respectively. This test is based on all 638 cases for participants who indicated that they would punish a Dictator Game donation and reject an Ultimatum Game offer.

### Statistical approach

We used multi-model selection with model averaging (Burnham & Anderson, 2002; Grueber *et al.* 2011) to compare the explanatory power of different input variables. Continuous input variables were standardised (Gelman, 2008) and binary input variables were centred, so estimates can be considered on the same scale. Under this approach, we first specify a global model, containing all fixed effects and interactions that were specified in the pre-registered predictions. All possible models deriving from this global model are compared, resulting in a top model set, which contains all the models that are within 2 AICc units of the ‘best’ model (that with the lowest AICc value). Parameter estimates are obtained by averaging across this top model set. This approach therefore incorporates the uncertainty over the true parameter estimate when many models have similar levels of support. All estimates reported here are full model averages, which provide conservative estimates for terms that are not included in all the top models. All data and code are available at Raihani & Bell (2017*b*) https://figshare.com/s/c5bbc8330551b14bc91e.

### Unplanned analysis

We report one unplanned analysis, motivated by comments from an anonymous reviewer, to establish whether the effect of paranoia on punishment decision in the Dictator Game was mediated by the participant's tendency to attribute harmful intentions. This was achieved by identifying participants who had participated in a previous Dictator Game reported in Raihani & Bell (2017*a*) where harmful intent attributions were recorded. See online Supplementary Information for full details.

## Results

### Supported predictions

#### P1: Dictator Game donations will be lower than Ultimatum Game offers

**Fig. 1.**
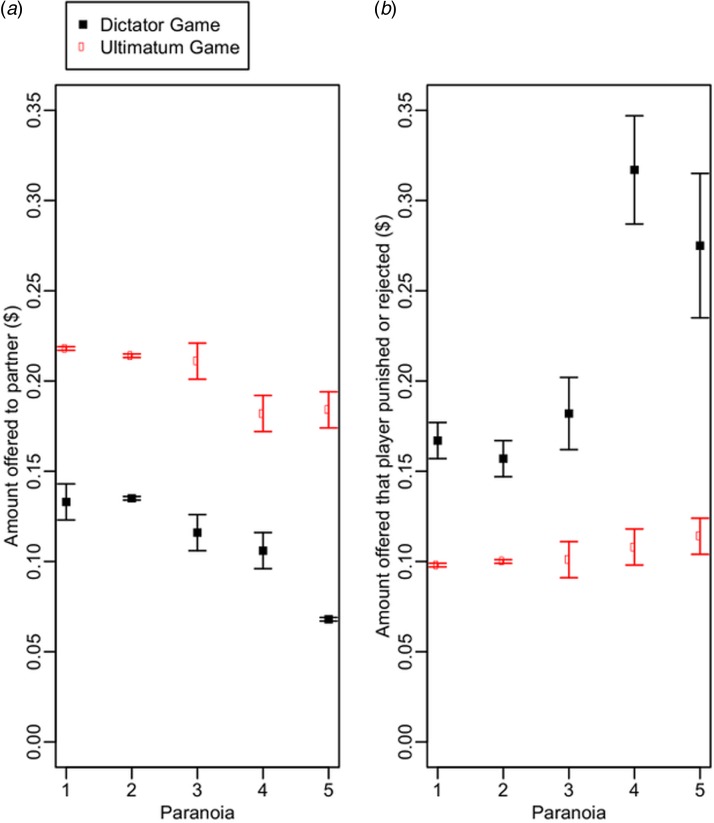
Effect of paranoia on (*a*) DG and UG offers made and (*b*) amount offered that the subject rejected (UG) or punished (DG). DG donations are shown in black and UG offers are shown in red. Data are raw means and standard errors and do not control for other terms included in the statistical models. Where no standard error bars are shown, this is because the standard error of the mean was 0.00 when rounded. For visualisation (and to calculate the raw means) paranoia was converted to a 5-level categorical variable, where 1 ⩽ 35, 35 < 2 ⩽ 60, 60 < 3⩽85, 85 < 4 ⩽ 110, and 110 < 5 ⩽ 160.

The mean dictator donation was $0.13 ± 0.00, compared with a mean Ultimatum Game offer of $0.21 ± 0.00. Thus, dictator donations were significantly lower than Ultimatum Game offers, as predicted (Wilcoxon signed rank test, *V* = 353 700, *p* < 0.001; Fig. 1*a*).

#### P4: Paranoia will be inversely associated with Dictator Game punishment threshold

A total of 685/1544 (44.4%) receivers chose to punish at least one dictator donation. The mean threshold below which dictator donations were punished was $0.18 ± 0.01 (i.e. ~33% of the stake). As predicted, paranoia resulted in lower punishment thresholds [estimate: −0.86, confidence interval (CI) −1.16 to −0.57; Table 1; Fig. 1*b*]. Women also had lower punishment thresholds than men and threshold decreased with age (Table 1). There were no meaningful effects of game order or wave on punishment thresholds, although we did find that failing comprehension checks was associated with a lowered punishment threshold (Table 1). Excluding failed comprehenders (*n* = 66; 9.6%) does not qualitatively change the results.

**Table 1.** Factors affecting punishment threshold

Parameter	Estimate	Unconditional s.e.	Confidence interval	Relative importance
*Intercept 1|2*	*−3.96*	*0.36*	(*−4.67 to −3.25*)	
*Intercept 2|3*	*−3.70*	*0.36*	(*−4.41 to −3.01*)	
*Intercept 3|4*	*−2.82*	*0.35*	(*−3.51 to −2.15*)	
*Intercept 4|5*	*−1.79*	*0.34*	(*−2.45 to −1.13*)	
*Intercept 5|6*	*−1.18*	*0.33*	(*−1.83 to −0.53*)	
*Intercept 6|7*	*−0.18*	*0.33*	(*−0.84 to 0.47*)	
Gender (female = 0)	0.36	0.14	(0.09 to 0.63)	1.00
Incorrect (all correct = 0)	−1.95	0.28	(−2.50 to −1.40)	1.00
Age	−0.74	0.15	(−1.03 to −0.46)	1.00
Paranoia	−0.86	0.15	(−1.16 to −0.57)	1.00
Order (DG first = 0)	−0.02	0.08	(−0.18 to 0.14)	0.29

Punishment threshold was parameterised as a 7-level ordinal categorical variable, where lower levels indicate increased willingness to punish higher DG offers. For binary input variables, the reference category is given in parentheses. All continuous input variables were standardized and binary input variables were centred. Thus, estimates can be interpreted as being on the same scale. Importance is the probability that the term in question is a component of the true best model.

#### P5: Paranoia will be associated with higher MAO in the Ultimatum Game

The mean MAO was $0.12 ± 0.00 (i.e. 24% of stake). Paranoia was associated with marginally higher MAO (estimate: 0.15; Fig. 1*b*), though the CIs associated with this estimate include zero (CI −0.07 to 0.37). Men demanded higher shares of the stake than women, as did participants that played the Ultimatum Game before the Dictator Game (Table 2). There were no meaningful effects of age or wave on MAO. Finally, failed comprehenders (*n* = 88, 5.7%) had lower MAOs than those who passed all comprehension checks. When failed comprehenders are excluded, then we detect a robust positive effect of paranoia on MAO (estimate: 0.22; CI 0.04–0.41).

**Table 2.** Factors affecting minimal acceptable offer (MAO) in the Ultimatum Game

Parameter	Estimate	Unconditional s.e.	Confidence interval	Relative importance
*Intercept 1|2*	*−1.38*	*0.16*	(*−1.70 to −1.07*)	
*Intercept 2|3*	*−0.21*	*0.16*	(*−0.51 to 0.10*)	
*Intercept 3|4*	*0.28*	*0.16*	(*−0.02 to 0.59*)	
*Intercept 4|5*	*0.73*	*0.16*	(*0.42 to 1.03*)	
*Intercept 5|6*	*1.89*	*0.16*	(*1.57 to 2.21*)	
*Intercept 6|7*	*4.61*	*0.28*	(*4.06 to 5.15*)	
Gender (female = 0)	0.24	0.09	(0.06 to 0.41)	1.00
Incorrect (all correct = 0)	−0.49	0.21	(−0.91 to −0.08)	1.00
Order (DG first = 0)	0.48	0.21	(0.30 to 0.66)	1.00
Paranoia	0.15	0.11	(−0.07 to 0.37)	0.82
Wave (wave 1 = 0)	0.01	0.05	(−0.08 to 0.11)	0.20
Age	0.00	0.04	(−0.07 to 0.08)	0.17

MAO was parameterised as a 7-level ordinal categorical variable, where higher levels indicate increased willingness to reject UG offers. For binary input variables, the reference category is given in parentheses. All continuous input variables were standardized and binary input variables were centred. Thus, estimates can be interpreted as being on the same scale. Importance is the probability that the term in question is a component of the true best model.

#### P6: Paranoia will be positively associated with willingness to punish at least one Dictator Game donation

Paranoia was positively associated with willingness to punish at least one dictator donation (estimate: 0.48, CI 0.27–0.70; online Supplementary Table S1). Furthermore, punishment was more common among males (estimate: 0.38; CI 0.16–0.59), among participants who failed comprehension check(s) (estimate: 1.38, CI 0.87–1.90), and among participants who played in the role of Ultimatum Game responder before they made their punishment decision in the Dictator Game (estimate: 0.62, CI 0.41–0.83). We detected no meaningful effects of age or wave on the willingness to punish (online Supplementary Table S1). Excluding participants who failed comprehension checks does not qualitatively change these results.

### Unsupported predictions

#### P2: Dictator Game donations will not vary with paranoia

Paranoia had a negative effect on dictator donations (estimate: −0.29, CI −0.49 to −0.10; Fig. 1*a*). In addition, male dictators made lower donations than females (estimate: −0.37, CI −0.56 to −0.18; Table 3). Older participants and those that played the Dictator Game before the Ultimatum Game made higher donations (Table 3). In a separate, unregistered analysis, we found a positive effect of paranoia on the tendency to fail at least one comprehension check (GLM, estimate = 0.02, CI 0.00–0.03). Thus, the negative effect of paranoia on dictator donations emerges even though failed comprehenders were more – not less – generous in the task. Excluding failed comprehenders from analyses does not change these results qualitatively.

**Table 3.** Factors affecting Dictator Game offer

Parameter	Estimate	Unconditional s.e.	Confidence interval	Relative importance
*Intercept 1|2*	*−0.22*	*0.22*	(*−0.66 to 0.22*)	
*Intercept 2|3*	*0.10*	*0.22*	(*−0.34 to 0.54*)	
*Intercept 3|4*	*0.27*	*0.22*	(*−0.17 to 0.71*)	
*Intercept 4|5*	*0.53*	*0.23*	(*−0.09 to 0.98*)	
*Intercept 5|6*	0.79	*0.23*	(*0.35 to 1.23*)	
*Intercept 6|7*	4.64	0.31	(*4.03 to 5.26*)	
Gender (female = 0)	−0.37	0.10	(−0.56 to −0.18)	1.00
Incorrect (all correct = 0)	0.89	0.27	(0.36 to 1.43)	1.00
Order (DG first = 0)	−0.20	0.10	(−0.39 to −0.02)	1.00
Age	0.40	0.10	(0.20 to 0.59)	1.00
Paranoia	−0.29	0.10	(−0.49 to −0.10)	1.00
Wave (1st wave = 0)	−0.03	0.07	(−0.17 to 0.11)	0.34

DG offer was parameterised as a 7-level ordinal categorical variable. For binary input variables, the reference category is given in parentheses. All continuous input variables were standardized and binary input variables were centred. Thus, estimates can be interpreted as being on the same scale. Importance is the probability that the term in question is a component of the true best model.

#### P3: Ultimatum Game offers will increase with paranoia

Paranoid participants made lower Ultimatum Game offers (estimate: −0.32, CI −0.53 to −0.11; Table 4; Fig. 1*a*). As with dictator donations, older participants made higher Ultimatum Game offers (estimate: 0.34, CI 0.11–0.56) but we detected no meaningful effects of gender or game order on offer (Table 4). There was no effect of failing a comprehension check on Ultimatum Game offers so we did not conduct further analyses where failed comprehenders were excluded.

**Table 4.** Factors affecting Ultimatum Game offer

Parameter	Estimate	Unconditional s.e.	Confidence interval	Relative importance
*Intercept 1|2*	*−2.43*	*0.26*	(*−2.93 to −1.92*)	
*Intercept 2|3*	*−2.22*	*0.26*	(*−2.72 to −1.71*)	
*Intercept 3|4*	*−1.77*	*0.25*	(*−2.26 to −1.27*)	
*Intercept 4|5*	*−1.36*	*0.25*	(*−1.85 to −0.87*)	
*Intercept 5|6*	*−0.50*	*0.25*	(*−0.98 to −0.02*)	
*Intercept 6|7*	*4.43*	*0.33*	(*3.79 to 5.07*)	
Age	0.34	0.11	(0.11 to 0.56)	1.00
Paranoia	−0.32	0.11	(−0.53 to −0.11)	1.00
Gender (female = 0)	−0.02	0.07	(−0.15 to 0.11)	0.26
Incorrect (all correct = 0)	−0.05	0.17	(−0.38 to 0.28)	0.24

UG offer was parameterised as a 7-level ordinal categorical variable. For binary input variables, the reference category is given in parentheses. All continuous input variables were standardised and binary input variables were centred. Thus, estimates can be interpreted as being on the same scale. Importance is the probability that the term in question is a component of the true best model.

#### P7: Paranoia will be positively associated with tendency to reject at least one Ultimatum Game offer

There was no meaningful effect of paranoia on tendency to reject at least one offer in the Ultimatum Game (estimate: 0.01, CI −0.13 to 0.15; online Supplementary Table S2). Men (estimate: 0.35, CI 0.08–0.62), and participants that played the Ultimatum Game first (online Supplementary Table S2), were more likely to reject at least one offer. By contrast, older participants and failed comprehenders were less likely to reject at least one offer (online Supplementary Table S2). Excluding failed comprehenders from this model does not qualitatively affect the results.

### Exploratory analysis results

#### P8: We do not know whether participants will be more willing to reject Ultimatum Game offers than they are to punish Dictator Game donations

Participants were more willing to punish dictator donations than they were to reject Ultimatum Game offers of the same proportional value (Wilcoxon signed rank test, *V* = 100 950, *p* = 0.03). Specifically, the average maximum dictator donation that was punished was $0.16 (30 ± 1% of the stake), whereas the mean maximum Ultimatum Game rejection was $0.13 (25 ± 1% of the stake).

### Unplanned analysis results

Punishment decisions were significantly mediated by a tendency to make harmful intent attributions although there was also a direct effect of paranoia. See online Supplementary Information for all details.

## Discussion

We used a large, non-clinical sample to explore the association between paranoia and (i) cooperation and (ii) willingness to punish in social interactions.

### Paranoia and cooperative behaviour

Previous work has suggested that reduced cooperation in paranoia could be explained by distrust (Fett *et al.* 2012; Ellett *et al.* 2013; Gromann *et al.* 2013; although see Fett *et al.* 2016). Our findings suggest that distrust might not be the sole explanation, as we found that paranoia predicted lower generosity in the Dictator Game, where trust is not a strategic consideration. Moreover, in the Ultimatum Game – where distrust should predict higher offers – we found that paranoia was unexpectedly associated with lower offers. Our results suggest that reduced cooperation in paranoia reflects increased self-interest. Importantly, self-interest here is used in the economic sense of maximising individual payoffs (rather than the everyday sense suggesting ‘selfishness’).

### Paranoia and punitive behaviour

We expected that paranoia would positively predict punitive tendency. Our data mostly support our predictions: paranoia positively predicted the tendency to punish at all in the Dictator Game, and to punish more generous donations. Moreover, in an unplanned mediation analysis, the effect of paranoia on willingness to punish dictator donations was mediated by a tendency to attribute harmful intentions, along with a direct effect of paranoia.

In the Ultimatum Game, paranoia only reliably predicted punitive tendency when participants that failed at least one comprehension check were excluded from the analyses. Furthermore, unlike the Dictator Game, the tendency to reject at least one offer in the Ultimatum Game was not predicted by paranoia. This discrepancy in the results might stem from the fact that rejecting any offer over $0.05 in the Ultimatum Game was costlier than punishing in the Dictator Game (which cost $0.05 regardless of the dictator's donation). Moreover, the fee to fine ratio of punishment was always 1:3 in the Dictator Game but was variable in the Ultimatum Game from 1:9 (in the case where responders rejected an offer of $0.05) to 1:1 (in the case where responders rejected an offer of $0.25). These differences in the cost of punishing and the potential efficacy of punishment across the two games might help explain the finding that players were more willing to punish dictator donations than to reject Ultimatum Game offers, and might also have reduced the size of any effect of paranoia on Ultimatum Game rejections.

### Implications for theories of paranoia

Our results suggest that the pattern of reduced cooperation in paranoia better reflects self-interest rather than distrust. Importantly, self-interest is a proximal motivation and various other processes may underlie it. For example, maximising immediate payoffs can manifest as being less willing to invest in social interactions (Stevens & Hauser, 2004) and may be an adaptive strategy when living in environments where resource availability is scarce and/or unpredictable (Frankenhuis *et al.* 2016). The fact that paranoia is associated with a history of serious adverse life events (Cristóbal-Narváez *et al.* 2016) would support this explanation. Alternatively, Andreoni (1990) and Gromann *et al.* (2013) have suggested that reduced cooperation in paranoia may result from reduced subjective rewards from social interactions. These explanations are not mutually exclusive. Previous research has suggested that adverse life events raise the risk of reward system dysregulation later in life (review in Howes & Murray, 2014) suggesting a potential proximal mechanism for the impact of distal developmental experiences. On the basis of non-clinical studies, Fehr & Schmidt (1999) have argued that altered fairness preferences can be drivers of reduced cooperation and this is a plausible but yet unexplored approach to paranoia.

The mediation analysis showed that the tendency to attribute harmful intent to partners, as measured on a previous task, was a factor motivating punishment decisions, alongside a direct effect of paranoia. It is possible that punitive behaviour in this study reflected increased levels of hostility (Coid *et al.* 2016). However, it is important to note that punishment as conceptualised here does not necessarily imply anti-social or aggressive tendencies. Indeed, willingness to invest in punishment can provide benefits in the form of increased within-group cooperation (Yamagishi, 1986) and we note that the factors that encourage punitive behaviour are likely to vary across different scenarios (see Raihani & McAuliffe, 2012; Bone & Raihani, 2015; Raihani & Bshary, 2015; Krasnow *et al.* 2016).

Freeman & Garety (2014) conceptualise paranoia as being maintained by a feedback loop of behaviour, interpretation and emotion, suggesting a dynamic system in which different motivations may apply depending on the incentive structure of specific situations. We found reduced cooperation and increased punitive behaviour in paranoia but suggest that the motivations are likely to be different in each case. We suggest here that models of paranoia need to include not only common factors but also the dynamics and contextual modifiers of social situations to fully understand how paranoia manifests in social behaviour. Considering that negative social interactions are themselves likely to act as maintaining factors for paranoia, identifying these cycles of maintenance may be important for identifying points of intervention.

### Limitations

The GPTS asks about paranoid ideation over the prior month and participants completed the experimental tasks in the subsequent weeks. It is possible that levels of paranoid thinking may have altered during this time, although given the good test-retest reliability of the measure (Green *et al.* 2008), we presume this effect would be small. Additionally, we recruited our sample from an online platform. Previous research has shown that online participants do not produce systematically different responses to other participants (Horton *et al.* 2011; Paolacci & Chandler, 2014; Hauser & Schwarz, 2016; Arechar *et al.* 2017). The current study supports this position. The mean and distribution of GPTS scores closely reflect findings from the original Green *et al.* (2008) validation study. Dictator Game donations were 23.6% of the stake compared with the mean of 28% reported in the Engel (2011) meta-analysis, and mean Ultimatum Game offers were 42%, compared with previously reported values of 30–40% (Camerer, 2003). Although stake sizes on MTurk are often smaller than those used in the laboratory, they are designed to reflect similar hourly rates of pay. Moreover, stake size has been found not to significantly affect behaviour in the Dictator Game or the Ultimatum Game (Camerer, 2003; Raihani *et al.* 2013). Although participants recruited online tend to be more representative than student samples or community samples from college towns (Buhrmester *et al*. 2011), online samples also tend to be younger, less-likely to report full-time employment, and more likely to experience social anxiety than the general population (Chandler & Shapiro, 2016) and this needs to be borne in mind when interpreting these results.

## Supplementary material

For supplementary material accompanying this paper visit https://doi.org/10.1017/S0033291717003075. click here to view supplementary material
