# Supplementary material for: Conflict and cooperation in paranoia: a large-scale behavioural experiment
Source: Psychol Med. 2017 Oct 17;48(9):1523–31. doi: 10.1017/S0033291717003075 (PMC6088528; doi:10.1017/S0033291717003075)
Supplement: Supplementary file 1 [file S0033291717003075sup.zip › S0033291717003075sup002.docx]

**Title:**

**Supplementary Information: Conflict and Cooperation in Paranoia: A Large-Scale Behavioural Experiment**

**Author names and affiliations:**

Nichola J. Raihani^a^, Vaughan Bell^b,c*^

a. Department of Experimental Psychology, University College London.

b. Division of Psychiatry, University College London.

c. South London and Maudsley NHS Foundation Trust

1. Statistical Information

2. The Green et al. (2008) Paranoid Thoughts Scale

3. Proposer and Dictator Game Instructions

4. Responder and Receiver Game Instructions

5. Table S1

6. Table S2

7. Information on subject naïveté and skepticism

8. Mediation analysis of punishment decisions

9. R code for mediation analysis

10. References

**1. Statistical Information**

We used multi-model inference with model averaging to determine the estimates associated with input variables of interest. Under this approach, a single global model is generated, which contains all explanatory terms of interest. Using the *dredge* function in the package MuMIn (Bartón, 2009), we then compare all possible submodels (i.e. all models containing every different possible combination of the terms included in the global model) and, from this process, derive a top model set. Models are compared to one another using an information-theoretic approach. Specifically, the Akaike Information Criterion, corrected for small sample sizes (AICc, Hurvich & Tsai, 1989) is used to compare the support generated for each model. AICc reflects the amount of variance explained by the candidate model, while also penalising the model for the inclusion of each explanatory term. Low AICc values indicate that the candidate model has greater support (i.e. explains more of the observed variance in the data) than any of the other candidate models – in other words, AICc values are inherently relative constructs. The top model set is the group of models that contains the best model (the model with the lowest AICc value) and all terms within 2 AICc units of this best model. To account for uncertainty over which of the top models is the true best model (given that they all have similar levels of support), we then average over this top model set to obtain model-averaged estimates and confidence intervals for the terms included in the top model set. Note that all continuous explanatory terms were standardized (by dividing by two standard deviations, Gelman 2008) and all binary explanatory terms were centred (by subtracting the mean). This means that estimates presented here are standardized and can thus be considered on the same scale. We report full model-averaged estimates and confidence intervals, which provide conservative estimates for terms that are not included in all models in the top model set.

**2. The Green et al. (2008) Paranoid Thoughts Scale**

To begin, **please enter your Amazon Mechanical Turk WorkerID** here:

(Please see below for where you can find your Worker ID.)

Your Worker ID starts with the letter A and has 12-14  letters or numbers. It is NOT your email address. If we do not have your correct Worker ID we will not be able to pay you.

Your WorkerID can be found on your dashboard page.

You are about to take part in an academic study which is run by the Raihani Lab, based at University College London. This project has been approved by the UCL Ethics Board project 3720/001.

By continuing, you are consenting to allow the Raihani Lab to use your responses in the study for academic purposes.

The purpose of this study is to understand people's behaviour. All data are anonymous (your name will not appear in any publication related to this study and will not be shared with any other parties). By completing this HIT you will be granted a qualification to participate in a subsequent HIT run by Raihani Lab in the future. You will be notified when the next HIT becomes available.

Please tick 'I agree' if you agree to these conditions. If you do not wish to participate, or if you change your mind during the course of the study, please close this window.

- I agree (1)

Please read each of the following statements carefully. They refer to thoughts and feelings you may have had about others **over the last month**. Think about last month and indicate the extent of these feelings from **1 (Not at all) to 5 (Totally).**

**Please complete both Part A and Part B.**

(N.B. Please do not rate items according to any experiences you may have had under the influence of drugs.)

**Part A:**

|  | Not at all (1) | 2 | 3 | 4 | Totally (5) |
| --- | --- | --- | --- | --- | --- |
| 1. I spent time thinking about friends gossiping about me. |  |  |  |  |  |
| 2. I often heard people referring to me. |  |  |  |  |  |
| 3. I have been upset by friends and colleagues judging me critically. |  |  |  |  |  |
| 4. People definitely laughed at me behind my back. |  |  |  |  |  |
| 5. I have been thinking a lot about people avoiding me. |  |  |  |  |  |
| 6. People have been dropping hints for me. |  |  |  |  |  |
| 7. I believed that certain people were not what they seemed. |  |  |  |  |  |
| 8. People talking about me behind my back upset me. |  |  |  |  |  |
| 9. I was convinced that people were singling me out. |  |  |  |  |  |
| 10. I was certain that people have followed me. |  |  |  |  |  |
| 11. Certain people were hostile towards me personally. |  |  |  |  |  |
| 12. People have been checking up on me. |  |  |  |  |  |
| 13. I was stressed out by people watching me. |  |  |  |  |  |
| 14. I was frustrated by people laughing at me. |  |  |  |  |  |
| 15. I was worried by people's undue interest in me. |  |  |  |  |  |
| 16. It was hard to stop thinking about people talking about me behind my back. |  |  |  |  |  |

**Part B:**

|  | Not at all (1) | 2 | 3 | 4 | Totally (5) |
| --- | --- | --- | --- | --- | --- |
| 1. Certain individuals have had it in for me. |  |  |  |  |  |
| 2. I have definitely been persecuted. |  |  |  |  |  |
| 3. People have intended me harm. |  |  |  |  |  |
| 4. People wanted me to feel threatened, so they stared at me. |  |  |  |  |  |
| 5. I was certain people did things in order to annoy me. |  |  |  |  |  |
| 6. I was convinced there was a conspiracy against me. |  |  |  |  |  |
| 7. I was sure someone wanted to hurt me. |  |  |  |  |  |
| 8. I was distressed by people wanting to harm me in some way. |  |  |  |  |  |
| 9.I was preoccupied with thoughts of people trying to upset me deliberately. |  |  |  |  |  |
| 10. I couldn't stop thinking about people wanting to confuse me. |  |  |  |  |  |
| 11. I was distressed by being persecuted. |  |  |  |  |  |
| 12. I was annoyed because others wanted to deliberately upset me. |  |  |  |  |  |
| 13. The thought that people were persecuting me played on my mind. |  |  |  |  |  |
| 14. It was difficult to stop thinking about people wanting to make me feel bad. |  |  |  |  |  |
| 15. People have been hostile towards me on purpose. |  |  |  |  |  |
| 16. I was angry that someone wanted to hurt me. |  |  |  |  |  |

**3. Proposer and Dictator Game Instructions**

To begin, please enter your Amazon Mechanical Turk WorkerID here:

(Please see below for where you can find your Worker ID.)

Your Worker ID starts with the letter A and has 12-19 letters or numbers. It is NOT your email address. If we do not have your correct Worker ID we will not be able to pay you.

You are about to take part in an academic study which is run by the Raihani Lab, based at University College London. This project has been approved by the UCL Ethics Board project 3720/001.   Please note that Raihani Lab does not use deception. All participants are real.   By continuing, you are consenting to allow the Raihani Lab to use your responses in the study for academic purposes.   The purpose of this study is to understand people's behaviour. All data are anonymous (your name will not appear in any publication related to this study and will not be shared with any other parties).   You will take part in two tasks.   You will participate with a different worker in each task.   Please tick 'I agree' if you agree to these conditions. If you do not wish to participate, or if you change your mind during the course of the study, please close this window.

- I agree (1)

**Task A**

{proposer giving info}

You are Player X. You have been allocated a bonus of $0.50.

Your partner is Player Y.

You can offer to share any amount of the bonus with the Player Y. However, Player Y can also choose whether to accept or refuse your offer.

If Player Y accepts your offer, then you both get the division you proposed.

If Player Y rejects your offer, then you both get nothing.

For example, if you offer $0.10 to Player Y and they accept the offer, then you will get $0.40 and Player Y will get $0.10.

However, if you offer $0.10 to Player Y and they reject the offer then you will both get $0.00.

Your worker ID will not be revealed to the other player and you will not find out their worker ID.

{question info} First, please answer two questions to ensure you have understood the game.

{P comp 1} Q1. If you offer $0.20 to Player Y and Player Y accepts your offer, what will your bonus be?

- $0.20 (1)
- $0.00 (2)
- **$0.30 (3)**

{Pcomp 2} Q2. If you offer $0.20 to Player Y and Player Y rejects your offer, what will your bonus be?

- $0.30 (1)
- **$0.00 (2)**
- $0.50 (3)

{Incorrect Q1} You answered question 1 incorrectly. Please try again.

Q1. If you offer $0.20 to Player Y and Player Y accepts your offer, what will your bonus be? Please provide your answer in the form $X.XX (i.e. numerical format) but do not include the '$' sign.

{Incorrect Q2}You answered question 2 incorrectly. Please try again.

Q2. If you offer $0.20 to Player Y and Player Y rejects your offer, what will your bonus be? Please provide your answer in the form $X.XX (i.e. numerical format)

{Decision} Please now indicate what share of the $0.50 you would like to offer to Player 2. Remember, if Player 2 accepts your offer, you both get the share that you propose. If Player 2 rejects your offer, you both get nothing.

- Offer $0.00 to Player 2 (keep $0.50 for myself) (1)
- Offer $0.05 to Player 2 (keep $0.45 for myself) (2)
- Offer $0.10 to Player 2 (keep $0.40 for myself) (3)
- Offer $0.15 to Player 2 (keep $0.35 for myself) (4)
- Offer $0.20 to Player 2 (keep $0.30 for myself) (5)
- Offer $0.25 to Player 2 (keep $0.25 for myself) (6)
- Offer $0.30 to Player 2 (keep $0.20 for myself) (7)
- Offer $0.35 to Player 2 (keep $0.15 for myself) (8)
- Offer $0.40 to Player 2 (keep $0.10 for myself) (9)
- Offer $0.45 to Player 2 (keep $0.05 for myself) (10)
- Offer $0.50 to Player 2 (keep $0.00 for myself) (11)

**Task B**

This is a different task to the previous task you just took part in. The rules of this task are different and you will interact with a different worker in this task.

You are Player E. You have been allocated $0.55 bonus.

Your partner is Player D. Player D has been allocated $0.05 bonus.

You can choose how much of your bonus to send to Player D. Player D has to accept any share of the bonus that you offer.

For example:

if you send $0.00, then you will keep $0.55 and Player D will get $0.05 (i.e. their initial allocation).

if you send $0.20, then you will keep $0.35 and Player D will get $0.25.

Your worker ID will not be revealed to the other player and you will not find out their worker ID.

First, please answer three questions to ensure you have understood the game.

{DG comp 1} Q1. How can you get the largest bonus in this task?

- By giving all the money to Player D (1)
- **By keeping all the money for myself (2)**
- It depends if Player D accepts my offer or not. (3)

{DG comp 2} Q2. Can Player D refuse your offer in this task?

- Yes: if Player D refuses my offer we will both get nothing (1)
- **No: in this task, Player D has to accept whatever I send them. (2)**

{DG Comp Q3} Q3. What amount would you have to send to Player D to ensure you both get the same bonus? (remember you have $0.55 and Player D has $0.05)

- **$0.25 (1)**
- $0.20 (2)
- There is no division that would result in equal bonuses (3)

{Incorrect Q1} You answered Q1 incorrectly. Please try again. Q1. What amount should you keep to get the largest bonus in this task? Please give your answer in the form $X.XX (i.e. numerical format)

{Incorrect Q2} You answered question 2 incorrectly. Please try again. Q2. Can Player D refuse your offer in this task? Please write yes / no in the box below.

{Incorrect Q3} You answered Q3 incorrectly. Please try again. Q3. What amount would you have to send to Player D to ensure you both get the same bonus? Please give your answer in the form $X.XX (i.e. numerical format)

**4. Responder and Receiver Game Instructions**

Persecutory Ideation DG / UG responder task

{turkid} To begin, please enter your Amazon Mechanical Turk WorkerID here:    (Please see below for where you can find your Worker ID.)    Your Worker ID starts with the letter A and has 12-14  letters or numbers. It is NOT your email address. If we do not have your correct Worker ID we will not be able to pay you.

{find dashboard} Your WorkerID can be found on your dashboard page.

{consent} You are about to take part in an academic study which is run by the Raihani Lab, based at University College London. This project has been approved by the UCL Ethics Board project 3720/001.   Please note that Raihani Lab does not use deception. All participants are real.   By continuing, you are consenting to allow the Raihani Lab to use your responses in the study for academic purposes.   The purpose of this study is to understand people's behaviour. All data are anonymous (your name will not appear in any publication related to this study and will not be shared with any other parties).   You will take part in two tasks.   You will participate with a different worker in each task.   Please tick 'I agree' if you agree to these conditions. If you do not wish to participate, or if you change your mind during the course of the study, please close this window.

- I agree (1)

**Task A**

{proposer giving info} You are Player X. You have been allocated a bonus of $0.00. Player Y is your partner in this task. Player Y has been given a bonus of $0.50 and can offer you any division of this bonus. You can choose whether to accept or refuse the offer made by Player Y.   If you accept Player Y's offer, then you both get the division proposed by Player Y. If you reject Player Y's offer, then you both get nothing. For example, if Player Y offers you $0.10 and you accept the offer, then you will get $0.10 and Player Y will get $0.40. However, if Player Y offers you $0.10 and you reject the offer then you will both get $0.00.    Your worker ID will not be revealed to the other player and you will not find out their worker ID.

{question info} First, we would like you to answer two questions to ensure you have understood the game.

{P comp 1} Q1. If Player Y offers you $0.20 and you accept this offer, what will your bonus be?

- **$0.20 (1)**
- $0.00 (2)
- $0.30 (3)

{Pcomp 2} Q2. If Player Y offers you $0.20 and you reject this offer, what will your bonus be?

- $0.30 (1)
- **$0.00 (2)**
- $0.50 (3)

{Incorrect Q1} You answered Q1 incorrectly - please try again. Q1. If Player Y offers you $0.20 and you accept this offer, what will your bonus be? Please provide your answer in the form $X.XX (i.e. numerical format) but do not include the '$' symbol.

{Incorrect Q2} You answered Q2 incorrectly - please try again. Q2. If Player Y offers you $0.20 and you reject this offer, what will your bonus be? Please provide your answer in the form $X.XX (i.e. numerical format) but do not include the '$' symbol.

{decide} Please indicate below, for the following offers made by Player Y, whether you would accept or reject the offer.  Remember, if you accept the offer, you both get the division proposed by Player Y. If you reject the offer, you both get nothing. Your responses here will be matched with the offer made by Player Y and will determine your bonus, so please answer carefully.

|  | I would accept (1) | I would reject (we both get $0.00) (2) |
| --- | --- | --- |
| Player Y offers $0.00 (and keeps $0.50) (1) |  |  |
| Player Y offers $0.05 (and keeps $0.45) (2) |  |  |
| Player Y offers $0.10 (and keeps $0.40) (3) |  |  |
| Player Y offers $0.15 (and keeps $0.35) (4) |  |  |
| Player Y offers $0.20 (and keeps $0.30) (5) |  |  |
| Player Y offers $0.25 (and keeps $0.25) (6) |  |  |
| Player Y offers $0.30 (and keeps $0.20) (7) |  |  |
| Player Y offers $0.35 (and keeps $0.15) (8) |  |  |
| Player Y offers $0.40 (and keeps $0.10) (9) |  |  |
| Player Y offers $0.45 (and keeps $0.05) (10) |  |  |
| Player Y offers $0.50 (and keeps $0.00) (11) |  |  |

**Task B** This is a different task to the previous task you just took part in. The rules of this task are different and you will interact with a different worker in this task.

{Dic B info} You are Player C. You have been allocated $0.05 bonus. Your partner is Player D. Player 1 has been allocated $0.55 bonus.

Player D can choose how much of their bonus to send to you. You have to accept any share of the bonus that Player D offers. For example: if Player D sends you $0.00, then you will get $0.05 (i.e. your original allocation) and Player D will keep $0.55. if Player D sends you $0.20, then you will get $0.25 and Player D will keep $0.35. Although you have to accept any share of the bonus that Player D offers, you can choose to pay $0.05 to reduce Player D's bonus by $0.15. Your worker ID will not be revealed to Player D and you will not find out their worker ID.

First, we would like you to answer three questions to ensure you have understood the game.

{DG comp 1} Q1. Can you refuse the offer made by Player D?

- Yes, if I refuse it we both get $0.00. (1)
- **No, I have to accept any share that Player D sends to me in this task. (2)**

{DG comp 2} Q2. If Player D sends you $0.00 and keeps $0.55, and you then choose to pay $0.05 to reduce Player 1's bonus (by $0.15), what will your remaining bonus be.

- **$0.00 (1)**
- $0.05 (2)
- $0.15 (3)

{DG comp 3} Q3. Regardless of how much Player D sends you, do you earn a larger bonus if you reduce Player D's bonus or do nothing?

- **I earn the most by doing nothing (1)**
- I earn the most by reducing Player D's bonus (2)

{Incorrect Q1} You answered question 1 incorrectly. Please try again. Q1. Can you refuse the offer made by Player D? Please type "yes" or "no" in the box below.

{Incorrect Q2} You answered question 2 incorrectly. Please try again. Q2. If Player D sends you $0.00 and keeps $0.55, and you then choose to pay $0.05 to reduce Player D's bonus (by $0.15), what will your remaining bonus be. Please give your answer in the form $X.XX (i.e. numerical format) but do not include the '$' symbol.

{Incorrect Q3} You answered question 3 incorrectly. Please try again. Q3. Regardless of how much Player D sends you, do you earn a larger bonus if you reduce Player D's bonus or do nothing?

{Decision} Please consider how you would behave in each of the following scenarios (corresponding to different sharing decisions made by Player D). Specifically, you should indicate whether you would pay $0.05 to reduce Player D's bonus by $0.15 in each scenario. Your responses here will be matched with the actual decision made by Player D and will determine your bonus, so please answer carefully. Please also note that negative payoffs for Player D are not possible.

|  | I will pay $0.05 to reduce Player D's bonus by $0.15 (1) | I will not pay $0.05 to reduce Player D's bonus by $0.15 (4) |
| --- | --- | --- |
| Player D sends you $0.00 and keeps $0.55 (1) |  |  |
| Player D sends you $0.05 and keeps $0.50 (2) |  |  |
| Player D sends you $0.10 and keeps $0.45 (3) |  |  |
| Player D sends you $0.15 and keeps $0.40 (4) |  |  |
| Player D sends you $0.20 and keeps $0.35 (5) |  |  |
| Player D sends you $0.25 and keeps $0.30 (6) |  |  |
| Player D sends you $0.30 and keeps $0.25 (7) |  |  |
| Player D sends you $0.35 and keeps $0.20 (8) |  |  |
| Player D sends you $0.40 and keeps $0.15 (9) |  |  |
| Player D sends you $0.45 and keeps $0.10 (10) |  |  |
| Player D sends you $0.50 and keeps $0.05 (11) |  |  |
| Player D sends you $0.55 and keeps $0.00 (12) |  |  |

{demographic info}Thanks - it's nearly the end! Before you go, it would be very helpful if you could answer some additional questions to help with our research. Unlike some other research labs, Raihani Lab does not use deception on MTurk. All participants are real. Nevertheless, for our own purposes, it is helpful to know to what extent you believed that the other player really existed.

|  | 1 - Very skeptical that other player was real (1) | 2 (2) | 3 (3) | 4 (4) | 5 -Very confident that other player was real (5) |
| --- | --- | --- | --- | --- | --- |
| Please choose one (1) |  |  |  |  |  |

Q36 To what extent have you participated in HITs similar to this before?

|  | 1 - Nothing like this scenario (1) | 2 (2) | 3 (3) | 4 (4) | 5 - Exactly like this scenario (5) |
| --- | --- | --- | --- | --- | --- |
| Please choose one (1) |  |  |  |  |  |

Q92 What is your age?

Q93 What is your gender

- Male (1)
- Female (2)

Q94 Which categories best describe you? You can select more than one option

- White (e.g. German, Irish, Italian, British etc) (1)
- Black or African American (e.g. Jamaican, Haitian, Nigerian etc) (2)
- American Indian or Alaska Native (e.g. Najavo Nation, Blackfeet Tribe, Mayan etc) (3)
- Asian (e.g. Chinese, Filipino, Asian Indian etc) (4)
- Native Hawaiian or Pacific Islander (e.g. Najavo Hawaiian, Samoan, Fijian etc) (5)
- Hispanic, Latino or Spanish Origin (e.g. Mexican, Mexican-America, Puerto-Rican, Cuban etc) (6)
- Middle Eastern or North African (e.g. Lebanese, Iranian, Egyptian, Moroccan etc) (7)
- Other (please give detail below) (8)

Q95 If you answered 'other', please provide some more info below.

Q96 Which categories best describe you?

- I was born in the US, so were both my parents and all my grandparents (1)
- I was born in the US, so were both of my parents, but at least one of my grandparents came from somewhere else (2)
- I was born in the US, but one of my parents was not (3)
- I was born in the US but both of my parents were not (4)
- I was not born in the US (5)
- Other (6)

Q97 If you answered that either you, your parents or your grandparents came from somewhere else, could you please provide some additional detail below.

Q26 Thanks for taking part - that's the end of the task.The decisions you made in this game will now be matched to the decisions made by other workers. Your bonus will depend on(i) how much Player Y offered you;(ii) whether you accepted Player Y's offer;(iii) how much Player D sent you; and(iv) whether you spent $0.05 to reduce Player D's bonus. Once the HIT is completed we will compute bonuses and pay all players. Bonuses will be paid within 10 days of the entire batch being completed. In order to receive your bonus, please be sure to enter the mystery word below in your HIT window before submitting your HIT.Thanks for playing!  Mystery word: squeak

{Decision} Please now indicate what share of the bonus you would like to send to Player D.

Reminder: the starting bonuses are $0.55 (you) and $0.05 (Player D). Remember, Player D must accept whatever division you propose.

- Send $0.00 to Player D (keep $0.55 for myself) (1)
- Send $0.05 to Player D (keep $0.50 for myself) (2)
- Send $0.10 to Player D (keep $0.45for myself) (3)
- Send $0.15 to Player D (keep $0.40 for myself) (4)
- Send $0.20 to Player D (keep $0.35 for myself) (5)
- Send $0.25 to Player D (keep $0.30 for myself) (6)
- Send $0.30 to Player D (keep $0.25 for myself) (7)
- Send $0.35 to Player D (keep $0.20 for myself) (8)
- Send $0.40 to Player D (keep $0.15 for myself) (9)
- Send $0.45 to Player D (keep $0.10 for myself) (10)
- Send $0.50 to Player D (keep $0.05 for myself) (11)
- Send $0.55 to Player D (keep $0.00 for myself) (12)

{Demographic Info} Thanks - it's nearly the end! Before you go, it would be very helpful if you could answer some additional questions to help with our research. Unlike some other research labs, Raihani Lab does not use deception on MTurk. All participants are real. Nevertheless, for our own purposes, it is helpful to know to what extent you believed that the other player really existed.

|  | 1 - Very skeptical that other player was real (1) | 2 (2) | 3 (3) | 4 (4) | 5 -Very confident that other player was real (5) |
| --- | --- | --- | --- | --- | --- |
| Please choose one (1) |  |  |  |  |  |

Q36 To what extent have you participated in HITs similar to this before?

|  | 1 - Nothing like this scenario (1) | 2 (2) | 3 (3) | 4 (4) | 5 - Exactly like this scenario (5) |
| --- | --- | --- | --- | --- | --- |
| Please choose one (1) |  |  |  |  |  |

Q37 What is your age?

Q38 What is your gender

- Male (1)
- Female (2)

Q29 Which categories best describe you? You can select more than one option

- White (e.g. German, Irish, Italian, British etc) (1)
- Black or African American (e.g. Jamaican, Haitian, Nigerian etc) (2)
- American Indian or Alaska Native (e.g. Najavo Nation, Blackfeet Tribe, Mayan etc) (3)
- Asian (e.g. Chinese, Filipino, Asian Indian etc) (4)
- Native Hawaiian or Pacific Islander (e.g. Najavo Hawaiian, Samoan, Fijian etc) (5)
- Hispanic, Latino or Spanish Origin (e.g. Mexican, Mexican-America, Puerto-Rican, Cuban etc) (6)
- Middle Eastern or North African (e.g. Lebanese, Iranian, Egyptian, Moroccan etc) (7)
- Other (please give detail below) (8)

Q30 Please specify where you are from if you selected 'other'

Q31 Which categories best describe you?

- I was born in the US, so were both my parents and all my grandparents (1)
- I was born in the US, so were both of my parents, but at least one of my grandparents came from somewhere else (2)
- I was born in the US, but one of my parents was not (3)
- I was born in the US but both of my parents were not (4)
- I was not born in the US (5)
- Other (6)

Q32 If you answered that either you, your parents or your grandparents came from somewhere else, could you please provide some additional detail below.

Q26 Thanks for taking part - that's the end of the task.The decisions you made in this game will now be matched to the decisions made by other workers. Additional information: Player D was shown your decision and was given the option to pay $0.05 to reduce your bonus by $0.15. Your bonus will therefore depend on (i) how much you sent to Player D and whether they chose to reduce your bonus on the basis of that decision; (ii) how much you offered to Player Y and whether or not they accepted or rejected the offer. Once the HIT is completed we will compute bonuses and pay all players. Bonuses will be paid within 10 days of the entire batch being completed. In order to receive your bonus, please be sure to enter the mystery word below in your HIT window before submitting your HIT. Thanks for playing!

 Mystery word: farmyard

If you would like to know more about this study and related work being carried out then please send an email to RaihaniLab@gmail.com with the subject line "tell me more". We will periodically update you on the outcome of the studies and happy to share published articles as and when they become available.

**5. Table S1.** Factors affecting willingness to punish at least 1 DG offer (1/0).

| **Parameter** | **Estimate** | **Unconditional**  **SE** | **Confidence Interval** | **Relative Importance** |
| --- | --- | --- | --- | --- |
| Intercept | -0.87 | 0.13 | (-1.14, -0.61) |  |
| Gender (female = 0) | 0.38 | 0.11 | (0.16, 0.59) | 1.00 |
| Incorrect (all correct = 0) | 1.38 | 0.26 | (0.87, 1.90) | 1.00 |
| Order (DG first = 0) | 0.62 | 0.11 | (0.41, 0.83) | 1.00 |
| Wave | -0.08 | 0.12 | (-0.31, 0.14) | 0.51 |
| Paranoia | 0.48 | 0.11 | (0.27, 0.70) | 1.00 |

Punishment was a binary response variable. For binary input variables, the reference category is given in parentheses. All continuous input variables were standardized and binary input variables were centered. Thus, estimates can be interpreted as being on the same scale. Importance is the probability that the term in question is a component of the true best model.

**6. Table S2.** Factors affecting willingness to reject at least one UG offer (1/0).

| **Parameter** | **Estimate** | **Unconditional**  **SE** | **Confidence Interval** | **Relative Importance** |
| --- | --- | --- | --- | --- |
| Intercept | 2.11 | 0.26 | (1.60, 2.61) |  |
| Gender (female = 0) | 0.35 | 0.14 | (0.08, 0.62) | 1.00 |
| Incorrect (all correct = 0) | -0.87 | 0.25 | (-1.35, -0.38) | 1.00 |
| Order (DG first = 0) | 0.59 | 0.14 | (0.32, 0.86) | 1.00 |
| Age | -0.32 | 0.13 | (-0.58, -0.06) | 0.51 |
| Wave | 0.03 | 0.09 | (-0.15, 0.21) | 0.27 |
| Paranoia | 0.01 | 0.07 | (-0.13, 0.15) | 0.21 |

Rejecting at least one UG offer was a binary response variable. For binary input variables, the reference category is given in parentheses. All continuous input variables were standardized and binary input variables were centered. Thus, estimates can be interpreted as being on the same scale. Importance is the probability that the term in question is a component of the true best model.

**7. Information about subject naiveté and skepticism**

| **Task** | **1**  **(not at all confident)** | **2** | **3** | **4** | **5**  **(extremely confident)** | **N** |
| --- | --- | --- | --- | --- | --- | --- |
| **Dictator/Proposer** | 201 | 188 | 268 | 383 | 488 | 1,528 |
|  | 13.2 % | 12.3 % | 17.5 % | 25.1 % | 31.9 % |  |
| **Receiver/Responder** | 186 | 185 | 281 | 388 | 511 | 1,511 |
|  | 12.0 % | 12.0 % | 18.1 % | 25.0 % | 32.9 % |  |

**Table S3. Subject responses to the question of how confident they were that they were playing against a real person.**

| **Task** | **1**  **(nothing like this)** | **2** | **3** | **4** | **5**  **(exactly like this)** | **N** |
| --- | --- | --- | --- | --- | --- | --- |
| **Dictator/Proposer** | 116 | 255 | 593 | 482 | 82 | 1,528 |
|  | 7.59 % | 16.7 % | 38.8 % | 31.5 % | 5.37 % |  |
| **Receiver/Responder** | 227 | 310 | 569 | 386 | 59 | 1,511 |
|  | 14.6 % | 20.0 % | 36.7 % | 24.9 % | 3.80 % |  |

**Table S4.** Subject responses to the question of whether they had participated in tasks similar to this previously.

**8. Mediation analysis of punishment decisions**

We tested to what extent punishment decisions in the Dictator Game were mediated by that individual’s tendency to ascribe harmful intentions to a partner (thank you to an anonymous reviewer for this point). In this study, we did not ask for attributions of intent but due to the participants from this study also having previously participated in the study published by Raihani and Bell (2017) we could test to what extent punishment decisions in the Dictator Game of this present study were mediated by that individual’s tendency to ascribe harmful intentions to a partner measured in a previous study (reported in Raihani & Bell 2017).

Data were available for 1,357 participants. As described in Raihani & Bell (2017), harmful intent attributions involved rating on a scale of 0-100 the extent to which the participant perceived that the dictator’s decision was motivated by a desire to reduce their bonus, with 0 being ‘not at all’ and 100 being ‘completely’. We previously showed that paranoia positively predicts harmful intent attribution and we do not reproduce those analyses here.

To determine the extent to which punishment decisions were mediated by prior harmful intent attributions (potentially reflecting a general tendency for harmful intent attributions), we ran two versions of each model (Table S6.1). In Model 1, we include the explanatory terms gender, order, wave, age and paranoia. In Model 2, we also include the term ‘harmful intent’. We can infer that the effect of paranoia on punishment decisions is (at least partially) mediated by harmful intent if the effect size associated with paranoia is smaller in Model 2 than Model 1 (Baron & Kenny, 1986). Note that the effect sizes for the models here do not match those presented in the main text as these models are run on a smaller subset of the data (as explained above).

**Table S5**. Mediation analysis of tendency to punish (1/0) in the Dictator Game.

| **Model without mediator** | **Parameter** | **Estimate** | **95 % CI** |
| --- | --- | --- | --- |
|  | Intercept | -0.81 | (-1.09, -0.53) |
|  | Gender | 0.35 | (0.13, 0.57) |
|  | Order | 0.62 | (0.39, -0.84) |
|  | Wave | -0.13 | (-0.40, 0.14) |
|  | Paranoia | 0.42 | (0.05-0.14) |
| **Model with mediator** | **Parameter** | **Estimate** | **95 % CI** |
|  | Intercept | -0.90 | (-1.25, 0.56) |
|  | Gender | 0.41 | (0.19, 0.64) |
|  | Order | 0.65 | (0.43, 0.88) |
|  | Wave | -0.12 | (-0.38, 0.14) |
|  | Harmful Intent | 0.55 | (0.32, 0.78) |
|  | Paranoia | 0.34 | (0.11, 0.56) |
|  | Age | -0.01 | (-0.11, 0.09) |

This table shows that the effect of paranoia on punishment tendency is partly but not wholly mediated by previous harmful intent attribution. This can be inferred because the effect size associated with paranoia in Model 2 is smaller than in Model 1 – but there is nevertheless still a robust positive effect of paranoia on tendency to punish in Model 2, even when harmful intent attribution is accounted for. Note that the estimates presented here are standardized and were generated using multi-model selection with model averaging (Grueber et al., 2011).

To further estimate the mediating effect of harmful intent attribution on decision to punish, we also ran a path analysis model using the package “lavaan” in R (Roseel 2012) to determine the direct effect of paranoia on punishment tendency; and the indirect that was mediated via harmful intent. Input variables to the models were standardized. R code is presented below. This analysis also supported the results above in showing that harmful intent partially mediated the effect of paranoia on decision to punish but that there is still an independent direct effect of paranoia on punishment decision. The direct effect of paranoia was estimated to be 0.08 $\pm$ 0.3 (p = 0.003) while the indirect effect of paranoia mediated by harmful intent was 0.02 $\pm$ 0.01 (p = 0.001). In total, the combined effect was 0.10 $\pm$ 0.03 (p < 0.001).

The results provide additional evidence that punishment decisions are mediated by perceptions of harmful intent and we would hypothesise based on the cumulative evidence that this relationship would be stronger when punishment decisions and harmful intent attributions are measured within the same task.

**9. R code for mediation analysis in lavaan**

################################################################

Import data

################################################################

rec<-read.csv("Receiver_workbook_analysis.csv", header=T)

names(rec)

################################################################

remove NAs

################################################################

rec5<-subset(rec, harm_me!="")

rec1<-subset(rec5, Paranoia!="")

################################################################

center / standardize input variables

################################################################

Gen<-ifelse(rec1$Gender=="Male",1,0);Gen

rec1$cGen<-Gen-(mean(Gen))

Wv<-ifelse(rec1$Wave=="2",1,0);Wv

rec1$cOrd<-rec1$order_f-(mean(rec1$order_f));cOrd

rec1$cWave<-Wv-(mean(Wv))

sdPar<-sd(rec1 $Paranoia)

sdA<-sd(rec1$Age)

rec1$zPar<-rec1$Paranoia/(sdPar+sdPar)

rec1$zAge<-rec1$Age/(sdA+sdA)

sdharm<-sd(rec1$harm_me)

rec1$zharm<-rec1$harm_me/(sdharm+sdharm)

################################################################

conduct path analysis

################################################################

model.one<-'zharm ~ zPar+zAge+cGen+cWave+cOrd

Pun_Dg ~ zharm + zPar+zAge+cGen+cWave+cOrd'

fit<-sem(model.one, data=rec1)

summary(fit, standardized=T, fit.measures=T, rsq=T)

model.two<- 'zharm ~a*zPar

Pun_Dg ~ b*zharm+ c*zPar

indirect := a*b

direct := c

total := c+ (a*b)'

fit2<- sem(model.two, data=rec1)

summary(fit2, standardized=T, fit.measures=T, rsq=T)

**10. References**

Bartón K. MuMIn: multi-model inference. R package version 1.0. 0 [computer program].

Gelman A. Scaling regression inputs by dividing by two standard deviations. *Statistics in Medicine*. 2008;27(15):2865-2873. doi:10.1002/sim.3107.

Hurvich CM, Tsai CL. Regression and time series model selection in small samples. Biometrika. 1989 Jun 1:297-307.

Raihani, N.J. & Bell, V. Paranoia and the social representation of others: a large scale game theory approach. Scientific Reports, 7: 4544.

Rosseel, Y. (2012). lavaan: An R Package for Structural Equation Modeling. Journal of Statistical Software, 48(2), 1-36. URL http://www.jstatsoft.org/v48/i02/.
