# Supplementary material for: Conflict and cooperation in paranoia: a large-scale behavioural experiment
Source: Psychol Med. 2017 Oct 17;48(9):1523–31. doi: 10.1017/S0033291717003075 (PMC6088528; doi:10.1017/S0033291717003075)
Supplement: Supplementary file 1 [file S0033291717003075sup.zip › S0033291717003075sup001.pdf]

AsPredicted
Pre-Registration made easy

Paranoid thoughts and social behaviour (#2636)

Author(s)
Nichola Raihani (UCL) - n.raihani@ucl.ac.uk
Vaughan Bell (UCL) - vaughan.bell@ucl.ac.uk

Created: 01/24/2017 06:47 AM (PT)
Public: 07/20/2017 01:21 AM (PT)

1) What's the main question being asked or hypothesis being tested in this study?

H1: Paranoia will be positively associated with tendency to reject offers in the UG.
H2: Paranoia will be positively associated with willingness to punish in the DG.
H3: Paranoia will be associated with higher MAO (minimal acceptable offer in the UG).
H4: Paranoia will be inversely associated with threshold to punish (in the DG).
H5: DG offers will not vary with paranoia.
H6: UG offers will increase with paranoia.
H7: DG donations will be lower than UG offers.
H8: We do not know whether participants will be more willing to reject UG offers than they are to punish DG donations.
We already have GPTS scores for 3225 subjects (collected via MTurk in December 2015). We will re-recruit participants from this sample (also via MTurk).

2) Describe the key dependent variable(s) specifying how they will be measured.

(1) Reject offer: binary response term indicating whether participant accepted (1) or rejected (0) UG offer.
(2) Punish dictator: binary response term indicating whether participant punished (1) or did not punish (0) dictator donation.
(3) MAO: smallest amount that responders accept in the UG. Ordinal categorical variable. Number of levels will depend on the observed variation in responses.
(4) Punishment threshold: lowest DG donation that participants will punish. Ordinal categorical variable; number of levels depend on the observed variation in responses.
(5) UG offer: ordinal categorical variable (with < 11 levels, as above) based on the amounts proposers offers to responders.
(6) DG donation: ordinal categorical variable (with < 11 levels) based on the amount dictators send to receivers.
(7) Difference: UG offer – DG donation (within-subject variable). Ordinal categorical variable.

3) How many and which conditions will participants be assigned to?

All participants will be assigned to all conditions.
1. UG responder
2. DG punisher
3. UG proposer
4. DG dictator

4) Specify exactly which analyses you will conduct to examine the main question/hypothesis.

For all analyses a model selection approach with model averaging will be used (described in Grueber et al. 2011). Input variables will be standardized. Zero-averaging will be used.

H1&2: Two GLMMs with (i) reject offer and (ii) punish dictator set as response terms, respectively. Explanatory terms: paranoia, age, gender, UG offer/dictator donation, paranoia x offer/donation interaction. Participant id will be included as a random term.
H3-6: Five clms (Christensen, 2015) with (i) MAO, (ii) punishment threshold; (iii) UG offer, (iv) DG donation and (v) difference set as response terms, respectively. Explanatory terms: paranoia, age, gender.
H7: Paired t-test of UG offer vs DG donation. Two-tailed.
H8: Paired t-test of MAO vs punishment threshold.

5) Any secondary analyses?

6) How many observations will be collected or what will determine sample size? No need to justify decision, but be precise about exactly how the number will be determined.

We aim at recruiting 3225 participants for each condition, though there will inevitable be attrition in the number of participants who respond to our request. We will first send an email to all participants who are eligible. Once the responses have slowed down to be < 10 per day, we will send another request email to the remaining subjects who have not responded. Once responses are < 2 per day, we will stop data collection.

7) Anything else you would like to pre-register? (e.g., data exclusions, variables collected for exploratory purposes, unusual analyses planned?)

Available at https://aspredicted.org/mc5vz.pdf
(Permanently archived at http://web.archive.org/web/*/https://aspredicted.org/mc5vz.pdf)

8) Have any data been collected for this study already?

No, no data have been collected for this study yet
